# Wrinkled Janus SMoSe–XS2 (X = Mo, W) heterostructures: coupling mechanical flexibility with enhanced HER

**DOI:** 10.3389/fchem.2025.1720281

**Published:** 2025-12-19

**Authors:** Jianping Li, Liqi Xiong

**Affiliations:** 1 Automotive & Transportation Engineering, Shenzhen Polytechnic University, Shenzhen, Guangdong, China; 2 School of Mechanical and Electronic Engineering, Nanjing Forestry University, Nanjing, China

**Keywords:** Janus transition metal dichalcogenides, in-plane heterostructure superlattice, mechanical properties, hydrogen evolution reaction, SMoSe–XS2

## Abstract

Janus transition-metal dichalcogenide (TMD) heterostructures offer unique opportunities for coupling mechanical flexibility with catalytic functionality. Molecular dynamics simulations reveal that in-plane SMoSe–XS_2_ (X = Mo, W) heterostructures spontaneously form sinusoidal superlattices driven by interfacial asymmetry, exhibiting over fivefold enhancement in fracture strain and pronounced size-dependent strength. The periodic profile of Armchair and Zigzag configurations highlights the intrinsic link between structure and mechanical response. Density functional theory calculations further demonstrate that the wrinkled interfaces significantly improve hydrogen evolution reaction (HER) activity, with Gibbs free energies as low as −0.18 eV, resulting from the upward shift of the Se and S *p*-band centers near the Fermi level, facilitating optimal H adsorption. This work establishes Janus TMD heterostructure superlattices as promising candidates for multifunctional applications integrating mechanical adaptability and catalytic efficiency.

## 1 Introduction

In recent years, two-dimensional (2D) materials have been intensively investigated, largely inspired by graphene, which exhibits remarkable physical characteristics (Geim and Novoselov, 2007). The presence of a Dirac cone in graphene enables ultrafast charge transport (Stoller et al., 2008; Wang et al., 2015), however, the zero bandgap significantly limits practical uses, particularly in field-effect transistors (Novoselov et al., 2016). To overcome this drawback, transition metal dichalcogenides (TMDs) have emerged as attractive candidates due to their high carrier mobility (Li et al., 2018). More importantly, their finite bandgaps provide access to diverse functionalities, including photocatalytic water splitting (Liang et al., 2019; Radisavljevic et al., 2011). A notable example is the Janus SMoSe monolayer, synthesized to demonstrate out-of-plane piezoelectricity arising from broken symmetry, with strong potential for spintronic devices (Liu et al., 2016). Thermal transport studies revealed that SMoSe and WS_2_ possess conductivities of approximately 51.27 W·m^–1^·K^−1^ and 47.90 W·m^–1^·K^−1^, respectively, both displaying distinct temperature dependence (Qin et al., 2022). Furthermore, SMoSe shows versatility in photocatalysis (Ren et al., 2020; Ren et al., 2025a), nanoelectronics (Ren et al., 2025b), and energy-conversion technologies (Luo et al., 2025). Beyond these, novel magnetic responses and unusual thermal behaviors have also been uncovered in a broader range of 2D systems (Lia et al., 2017; Torun et al., 2023; Zhang et al., 2020).

For 2D materials, the effective strength relevant for engineering applications is determined primarily by fracture toughness rather than by the intrinsic bond strength of atoms (Ren et al., 2025c; Ren et al., 2025d). Graphene, for instance, exhibits brittle failure, with a measured fracture toughness close to 4.0 MPa (Cadelano et al., 2010). Interestingly, some layered systems display a negative Poisson’s ratio, such as δ-SiSe (−0.29) (Ren et al., 2022a), Li_2_B_12_ (−0.03) (Ren et al., 2022b), B_4_N (−0.02) (Wang et al., 2019), and δ-CS (−0.19) (Sun and Schwingenschlögl, 2020), which originates from their unique lattice geometry. These auxetic structures hold potential in advanced composites and aerospace technology. Moreover, many 2D systems show remarkable strain-tunable thermal and electronic responses (Huang L. et al., 2025; Huang W. et al., 2025; Ren and Sun, 2025). To broaden their functions, researchers have developed heterostructures. Van der Walls stacks with type-II band alignment, for example, enable efficient charge separation and extend carrier lifetime (Yan et al., 2020). In contrast, in-plane heterostructures rely on covalent connections. A notable case is the MoS_2_/WSe_2_ lateral junction, which was recently fabricated (Chiu et al., 2015). Its fracture resistance can be adjusted by the interface design, thermal environment, or preexisting cracks (Ren et al., 2023a). Similarly, Janus SMoSe/WS_2_ in-plane hybrids have been synthesized (Trivedi et al., 2020), showing intrinsic thermo-mechanical coupling at the interface (Ren et al., 2022c). Even more, ordered lateral superlattices have been experimentally realized, such as WSe_2_/WS_2_ (Wu et al., 2019), exhibiting diode-like transport with rectification ratios up to 10^5^. These artificial lattices have attracted widespread interest due to emergent phenomena including mini-Dirac cones (Li et al., 2015), Mott insulating states (Zhao and Schwingenschlögl, 2021), reduced lattice thermal conductivity (Yin et al., 2021) and moiré exciton bands (Torun et al., 2018). Specifically, the Janus SMoSe/WS_2_ superlattice band alignment can switch from type-I to type-II depending on intrinsic structural parameters, making it suitable for photocatalytic water splitting (Zhang et al., 2023). Its electronic states can also be tuned effectively by external strain or electric fields (Shu et al., 2025). Despite these advances, the mechanical consequences of lateral superlattice architectures remain insufficiently explored. In particular, the intrinsic interfacial bending in Janus SMoSe/WS_2_ heterostructures largely determines their strength and could provide valuable guidelines for device design. Yet, the bending behavior itself has not been systematically investigated.

In this study, we predict that the in-plane Janus SMoSe–XS_2_ (X = W, Mo) heterostructure superlattice remains stable, exhibiting a nearly sinusoidal configuration arising from a well-matched interface. Furthermore, its adjustable mechanical responses are examined through molecular dynamics simulations by varying the dimensions of the SMoSe and XS_2_ domains, revealing an exceptionally stretchable nature.

## 2 Geometric structure and computational methods

In this study, molecular dynamics calculations were performed using the Large-scale Atomic/Molecular Massively Parallel Simulator (LAMMPS) (Plimpton, 1995). Atomic configurations were visualized through the OVITO software package (Stukowski, 2009). The in-plane SMoSe–WS_2_ heterostructure was modeled with the Stillinger–Weber (SW) potential formulated by Jiang, which has been widely applied to transition metal dichalcogenides due to its reliable description of structural and elastic behavior (Jiang, 2018; Jiang et al., 2013). The Stillinger–Weber (SW) potential was parameterized using a valence force field (VFF) framework (Jiang, 2015). Previous studies have demonstrated that both the VFF and SW models can accurately reproduce the experimentally measured phonon spectrum of MoS_2_ (Wakabayashi et al., 1975). Therefore, the molecular dynamics simulations employed in this work, which are based on the SW potential, are regarded as reliable. Besides, the stress–strain response and Young’s modulus of MoS_2_ derived from this potential agree well with experimental measurements (Bertola et al., 2011; Cooper et al., 2013), confirming its suitability for mechanical investigations of these layered crystals.

**FIGURE 1:**
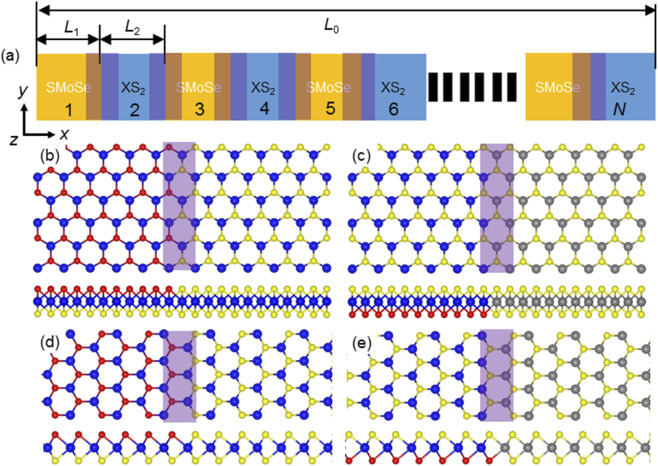
**(a)** Schematic illustration of the in-plane Janus SMoSe–XS_2_ heterostructure superlattice, assembled with four interface configurations: **(b)** A1, **(c)** A2, **(d)** Z1, and **(e)** Z2. The spheres in blue, pink, grey and yellow denote Mo, Se, W and S atoms, respectively.

The SMoSe–XS_2_ lateral superlattice was designed as illustrated in Figure 1a, where the number of SMoSe and XS_2_ segments is determined by the parameter *N*. Periodic boundary conditions were imposed along the *x*, *y*, and *z* directions. The characteristic lengths of the SMoSe, XS_2_, and heterostructure domains were denoted as *L*_1_, *L*_2_, and *L*_0_, respectively. Different heterostructure configurations were considered, as shown in Figures 1b–e: armchair SMoSe/MoS_2_ (A1), armchair SMoSe/WS_2_ (A2), zigzig SMoSe/MoS_2_ (Z1), and zigzig SMoSe/WS_2_ (Z2). Recent experiments have demonstrated the successful synthesis of lateral MoSSe/WSSe heterostructures featuring zigzag interfaces via chemical vapor deposition (CVD) (Trivedi et al., 2020). In that work, triangular MoSe_2_ monolayers were first grown, followed by epitaxial growth of WSe_2_ from the one-dimensional edges of the MoSe_2_ domains. Although MoSSe/WSSe heterostructures with armchair interfaces have not yet been experimentally realized, previous studies have shown that the orientation of 2D material domains–zigzag or armchair–can be precisely controlled through growth–etching–regrowth strategies in CVD processes (Huang et al., 2014; Zhang et al., 2018; Zhou et al., 2018). These advances suggest that catalytic applications based on MoSSe/WSSe heterostructures are likely to become experimentally accessible in the near future. First, the SMoSe–XS_2_ system was optimized at 300 K for 10 ps using the NPT ensemble (isothermal–isobaric conditions). Subsequently, the system was equilibrated for 2,000 ps under the NVT ensemble with a Nosé–Hoover thermostat. Finally, a 2000 ps relaxation was performed using the NVE ensemble (constant volume and energy). During this stage, both the total energy and temperature exhibited stable fluctuations, confirming that the system had reached equilibrium. The effective layer thickness of the system was set to 0.61 nm (Jiang et al., 2013).

For the mechanical assessment, uniaxial stretching was applied at a strain rate of 2 × 10^8^ s^–1^ in a specified direction, implemented through the fix/deform command in LAMMPS (Plimpton, 1995). The system temperature was maintained at 300 K, and zero pressure was applied along the *y*-axis during tensile deformation. To investigate the mechanical behavior of the SMoSe–XS_2_ heterostructure superlattice, the normal stress was tracked throughout the simulations. The atomic virial stress of Se, S, Mo, and W atoms is determined by Equation 1

$$\sigma_{i}=\frac{1}{\Omega_{i}}\left[m_{i}v_{i}⊗v_{i}+\frac{1}{2}\sum_{j\neq i}F_{ij}⊗r_{ij}\right]$$ (1)

Here, $\Omega_{i}$ , *m*_*i*_ and *v*_*i*_ are the volume correspond to the mass, velocity vector, and spatial volume of atom *i*, respectively. The interatomic force exerted by atom *j* on atom *i* is denoted as *F*_*ij*_, and the distance between these atoms is *r*_*ij*_. The symmetric stress tensor (*σ*_*i*_) consists of components *σ*_*xx*_, *σ*_*yy*_, *σ*_*zz*_, *σ*_*xz*_, *σ*_*xy*_ and *σ*_*yz*_; however, for a two-dimensional system, *σ*_*xz*_, *σ*_*yz*_ and *σ*_*zz*_ are negligible. Additionally, each atom’s volume is calculated as the initial relaxed system volume divided by the total atom count.

As for simulations of Gibbs free energy, density functional theory (DFT) calculations were carried out using the Vienna *ab initio* simulation package (VASP). The projector augmented-wave (PAW) method was applied to describe the interaction between core and valence electrons. Exchange–correlation effects were treated within the generalized gradient approximation (GGA) framework, employing the Perdew–Burke–Ernzerhof (PBE) functional. To account for long-range van der Waals interactions, Grimme’s DFT-D3 correction was included. The plane-wave cut-off energy was set to 550 eV, and a 15 × 15 × 1 Monkhorst–Pack *k*-point mesh was used to sample the Brillouin zone. A vacuum layer of 25 Å was introduced to prevent spurious interactions between periodic images and the total energy convergence threshold was set to 10^−5^ eV.

## 3 Results and discussion

The optimized lattice parameters of SMoSe, WS_2_ and MoS_2_ monolayers are 3.228 Å, 3.175 Å and 3.180 Å, respectively, as determined in our earlier study through first-principles calculations (Ren et al., 2022c). The lattice mismatch (*ϕ*) at the SMoSe–WS_2_ and SMoSe–MoS_2_ interface is approximately 1.6% and 0.15%, respectively, which is obtained as Equation 2:

ϕ=2aSMoSe – aXS2/(aSMoSe+a)XS2 (2)

where *a*_SMoSe_ and *a*_XS2_ demonstrate the lattice parameters of the SMoSe and XS_2_ monolayers. Then, we further calculated the binding energy (*E*_b_) of the A1, A2, Z1, and Z2 stacking SMoSe–WS_2_ and SMoSe–MoS_2_ interface by Equation 3:

$$E_{b}=E_{\text{SMoSe}}+E_{\text{XS}2}\;–\;E_{\text{SMoSe}–\text{XS}2}$$ (3)

where *E*_SMoSe_, *E*_XS2_ and *E*_SMoSe–XS2_ represent the energy of the SMoSe, XS_2_ and SMoSe–XS_2_ heterostructure, respectively. Then, the binding energy A1, A2, Z1 and Z2 configuration is calculated as −0.24 eV·Å^–1^, –0.18 eV·Å^–1^, –0.21 eV·Å^–1^ and –0.26 eV·Å^–1^, respectively, which is lower than that of the reported graphene/biphenylene interface (about −0.14 eV·Å^–1^) (Ren et al., 2022d). The obtained binding energy also demonstrates the stability of the studied SMoSe–XS_2_ (X = W, Mo) system.

Based on this, we constructed self-assembled superlattices with A1, A2, Z1, and Z2 stacking configurations. For the A1 and A2 models, the starting length and width are 620.168 Å and 51.265 Å, respectively. In contrast, the Z1 and Z2 configurations possess initial dimensions of 716.328 Å in length and 52.397 Å in width. Periodic boundary conditions are imposed along the *x*, *y*, and *z*-axes. In the present study, the overall dimensions of the SMoSe–WS_2_ superlattice are kept constant, while the lengths of the SMoSe and WS_2_ regions are treated as adjustable parameters.

**FIGURE 2:**
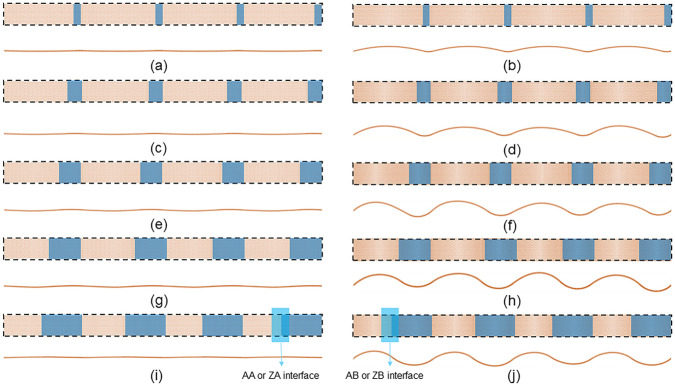
The optimized configurations are illustrated in both top and side views: **(a,c,e,g,i)** correspond to the A1 heterostructure, while **(b,d,f,h,j)** represent the A2 heterostructure. The ratios of *L*_1_ to *L*_2_ are set as **(a,b)** 1/9, **(c,d)** 2/8, **(e,f)** 3/7, **(g,h)** 4/6, and **(i,j)** 5/5, respectively.

Firstly, we examine how the length ratio *L*_1_/*L*_2_ influences the optimized geometry. After full relaxation, the equilibrium structures of the A1 and A2 SMoSe–WS_2_​ superlattice under varying *L*_1_/*L*_2_ are displayed in Figure 2. The A1 configuration remains nearly flat, maintaining a smooth monolayer surface, as illustrated in Figures 2a,c,e,g,i. In contrast, the A2 arrangement exhibits periodic folding driven by interfacial curvature, as depicted in Figures 2b,d,f,h,j. Notably, when *L*_m_/*L*_n_ approaches unity, the A2 phase evolves into a highly ordered sinusoidal sheet, as shown in Figure 2j. Such an intrinsic morphology differs fundamentally from previously reported wrinkled or folded monolayers induced by external mechanical strain (Li et al., 2015; Song et al., 2016; Yang et al., 2012). A similar effect is observed in the heterostructure superlattice formed with a zigzag interface.

**FIGURE 3:**
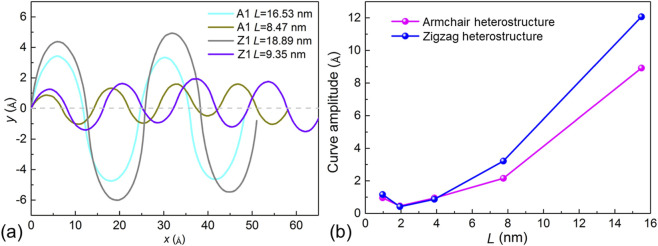
**(a)** The Fourier-fitted profile of the structural evolution in the SMoSe–WS_2_ superlattice and **(b)** the corresponding amplitude plotted as a function of *L*.

Inspired by the intriguing spontaneous bending observed in the SMoSe–XS_2_ superlattice, we investigated the quantitative features of its trajectory. To examine the tunable characteristics of this system, the lengths of the SMoSe and XS_2_ segments were constrained such that *L* = *L*_1_ = *L*_2_, as illustrated in Figure 1. The number of periods *N* was chosen as 4, 8, 16, 32, and 64, corresponding to lengths of 16.53 nm, 8.47 nm, 4.29 nm, 2.86 nm, and 1.87 nm, respectively, in both A1 and A2 superlattices. For the Z1 and Z2 configurations, the lengths are 18.89 nm, 9.35 nm, 5.45 nm, 3.25 nm, and 2.24 nm, respectively. The atomic coordinates along the *z*-axis were extracted and averaged, with the intermediate positions taken as zero. This allowed us to construct the trajectory of the superlattice. Curve fitting revealed that the side-view profiles of the relaxed structures conform remarkably well to a Fourier form. The fitted Fourier expressions for the armchair and zigzag SMoSe–XS_2_ superlattices with varying *L* are displayed in Figure 3a.

As shown in Figure 3b, the amplitude of the sinusoidal bending increases with larger *L*. This trend arises because each SMoSe or XS_2_ segment is subject to opposing intrinsic stresses on its boundaries. When the segment length *L*_1_ (or *L*_2_) is too short, the section resists bending, thereby reducing the wave amplitude. Additionally, the trajectory functions in Figure 3a can be represented as *y* = *a*sin(*bx*) + *c*cos(*dx*) + *e*, and the corresponding fitting parameters are summarized in Table 1.

**TABLE 1:** The extracted values describing the motion functions of the SMoSe–XS_2_ heterostructure superlattice.

​	*a*	*b*	*c*	*d*	*e*
A2 *L* = 16.53	0.78	0.35	−4.45	0.36	0.085
A2 *L* = 8.47	0.92	0.56	−0.85	0.56	0.063
Z2 *L* = 18.89	−4.35	0.33	−4.52	0.38	−0.021
Z2 *L* = 9.35	0.87	0.42	−1.45	0.42	−0.018

**FIGURE 4:**
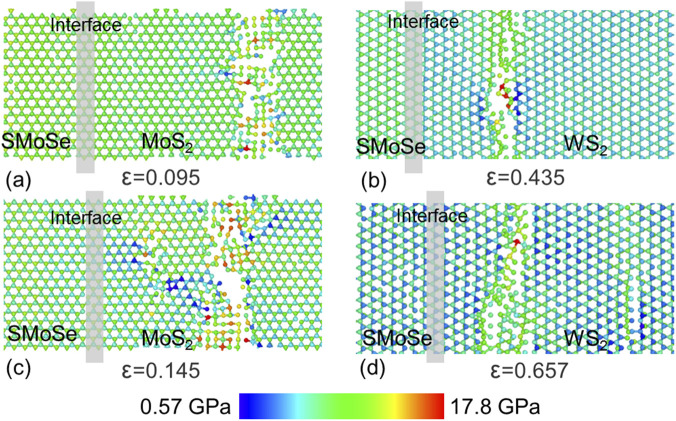
The crack nucleation of SMoSe–XS_2_ heterostructure superlattice with **(a,c)** A1 and **(b,d)** A2 interfaces. *L* is 16.53 nm and 18.89 nm, respectively, in **(a,b)** and **(b,d)**.

The fracture response of SMoSe and XS_2_ monolayers was evaluated. Under uniaxial loading along the armchair orientation, SMoSe fails at a strain of 0.125 with a breaking stress of 13.31 GPa, whereas MoS_2_ (or WS_2_) reaches 0.140 (or 0.151) strain and 19.85 GPa or (20.11 GPa) stress. When stretched in the zigzag orientation, SMoSe ruptures at 0.140 strain with 12.36 GPa stress, while MoS_2_ (or WS_2_) endures up to 0.130 (or 0.145) strain and 17.06 GPa MoS_2_ (or 18.56 GPa) stress. These results clearly show that XS_2_ exhibits superior strength compared with SMoSe. Consequently, in the SMoSe–XS_2_ superlattice, the earliest cracks form within the SMoSe domain, as illustrated in Figure 4. Figures 4a–d show the crack nucleation for heterostructures with A1, A2, Z1, and Z2 interfaces. Moreover, tensile loading along the zigzag axis produces greater atomic stress and initiates longer cracks. In addition, compared with A1 (or Z1) stacking, the A2 (or Z2) configuration demands higher critical stress before fracture occurs.

**FIGURE 5:**
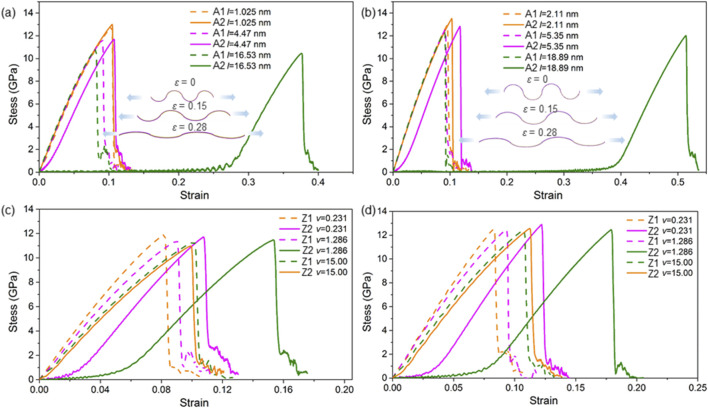
The stress–strain responses of the SMoSe–XS_2_ heterostructure superlattice are shown for **(a,c)** armchair and **(b,d)** zigzag interfaces with varying **(a,b)** lengths *L* and **(c,d)** ratio of *L*_1_/*L*_2_; the inset shows the side-view schematic illustrating the structural evolution of the A2 heterostructure under uniaxial tension.

To explore the strain–stress characteristics of the SMoSe–XS_2_ heterostructure superlattice, we analyzed how the armchair and zigzag configurations respond under external uniaxial strain. The nonlinear elastic behavior is illustrated in Figure 5. Notably, the fracture strength of the A1 (or Z1) and A2 (or Z2) SMoSe–XS_2_ superlattices is similar when *L* equals 1.02 nm (or 1.25 nm). In contrast, the tensile failure process of the A2 and Z2 configurations is significantly extended, as depicted in Figures 5a,b. Prior to reaching strains of 0.29 and 0.41, respectively, the stress levels in these structures remain comparatively low. This behavior arises because the relaxed state of A2 and Z2 superlattices adopts a bent geometry, which must be flattened before catastrophic fracture occurs, as shown as insets of Figures 5a,b. Additionally, the fracture stress of the SMoSe–XS_2_ heterostructures exceeds that of the MoS_2_/WS_2_ system (12.01 GPa along the zigzag direction and 12.68 GPa along the armchair direction) for *N* = 4 (Qin et al., 2019). Furthermore, the fracture strain for *N* = 4 is significantly higher than that of the MoS_2_/WS_2_ heterostructure (10% along the zigzag direction and 9.8% along the armchair direction). In addition, the stress–strain behavior of the A1 heterostructure superlattice with *L* = 1.14 nm was evaluated using DFT. Besides, the stress–strain behavior of the Z1 and Z2 heterostructure superlattice with different *L*_1_/*L*_2_ is also calculated as Figures 5c,d, which still presents the enhanced mechanical properties of SMoSe components. It is worth noting that the enhanced fracture strain also larger than that of investigated MoSSe/WSSe heterostructure (about 0.32) (Ren et al., 2023a), Si-Ge heterostructure (about 0.30) (Zhao et al., 2024), SiC grain boundaries (about 0.34) (Ren et al., 2023b).

**FIGURE 6:**
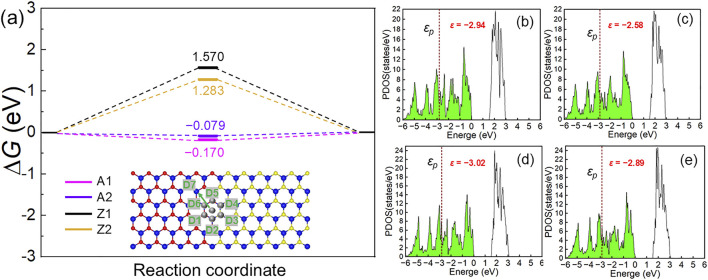
The calculated Gibbs free energy of **(a)** SMoSe–XS_2_ heterostructure interface; the projected *p*-orbital density of states of S or Se atoms for H adsorption sites of **(b)** A1, (B) A2, (B) Z1 and (B) Z2 SMoSe–XS_2_ heterostructures.

Beyond the obtained mechanical robustness, these SMoSe–XS_2_ heterostructures also exhibit intriguing electronic characteristics that may influence their catalytic behavior. Therefore, to further explore their functional potential, we investigated their hydrogen evolution reaction (HER) activity. The symmetric regions (D1–D7, shown as inset of Figure 6a) near the interface were examined to identify the most active sites. The most favorable H* adsorption occurs at D7 in the A1, A2, Z1 and Z2 configurations, decided by the lowest binding energies as −0.45 eV, −0.31 eV, 2.10 eV and 1.71 eV, respectively, which is calculated by Equation 4:

$$E_{b}=E_{H+\text{SMoSe}–\text{XS}2}+E_{\text{SMoSe}–\text{XS}2}\;–\;E_{H}$$ (4)

where *E*_H*+*SMoSe–XS2_, *E*_SMoSe–XS2_ and *E*_H_ represent the total energy of the H absorbed SMoSe–XS_2_ system, pure SMoSe–XS_2_ and H, respectively.

The calculated Gibbs free energies (Δ*G*) for HER are −0.170, −0.079, 1570 and 1.283 eV for the A1, A2, Z1 and Z2 configurations, respectively. One can see that the wrinkled configurations (A2 and Z2) exhibit enhanced HER activity comparing with that of flat structure (A1 and Z1). The enhanced HER catalytic activity of the reverse SMoSe–XS_2_ heterostructure can be further understood from the projected band center (*ε*_*p*_) analysis. The formation of Se–H and S–H bonds is primarily governed by the p orbitals of Se and S atoms at the active sites, therefore, the position of the *p* orbital relative to the Fermi level is critical for H* adsorption. As shown in Figures 6b–e, the density of states (DOS) of the atoms at the active sites reveals that the –|*ε*_*p*_| of the A2 and Z2 configurations configuration lies closer to the Fermi level than that of A1 and Z1 one, explaining the stronger H adsorption on the former structures. Thus, such wrinkled SMoSe–XS_2_ interface also can improve the HER activity.

## 4 Conclusion

In summary, this work reveals that Janus SMoSe–XS_2_ (X = Mo, W) heterostructures can spontaneously form sinusoidal superlattices due to interfacial asymmetry, leading to exceptional mechanical flexibility and stability. The resulting wrinkled configurations exhibit a substantial increase in fracture strain and tunable strength, governed by periodic structural modulation. Beyond mechanical robustness, the same interfacial characteristics enhance the catalytic performance for hydrogen evolution reaction (HER), with the A2 interface showing nearly ideal hydrogen adsorption free energy. The improved activity originates from the optimized electronic structure and the *p*-band alignment of S and Se atoms near the Fermi level. These findings demonstrate that structural asymmetry in Janus TMD heterostructures can synergistically engineer both mechanical and catalytic functionalities, offering a viable strategy for designing multifunctional 2D materials.

## Data Availability

The raw data supporting the conclusions of this article will be made available by the authors, without undue reservation.
